# Cytoskeletal alterations in neuronal cells implicate *Toxoplasma gondii* secretory machinery and host microRNA-containing extracellular vesicles

**DOI:** 10.1038/s41598-025-96298-8

**Published:** 2025-04-12

**Authors:** Thomas Mazza, Morteza Aslanzadeh, Lïse Berentsen, Franziska Bonath, Marc R. Friedländer, Antonio Barragan

**Affiliations:** 1 Department of Molecular Biosciences, The Wenner-Gren Institute, Stockholm University, Stockholm, Sweden; 2 Science for Life Laboratory, Department of Molecular Biosciences, The Wenner-Gren Institute, Stockholm University, Stockholm, Sweden; 3 Department of Gene Technology, School of Engineering Sciences in Chemistry, Biotechnology and Health, Kungliga Tekniska Högskolan, Stockholm, Sweden

**Keywords:** Neuron, Extracellular vesicle, microRNA, Host–pathogen interaction, Apicomplexan parasites, Cytoskeleton, Parasitology, Pathogenesis

## Abstract

The widespread protozoan *Toxoplasma gondii* chronically infects neural tissue in vertebrates and is linked to various neurological and neuropsychiatric disorders in humans. However, its effects on sparsely infected neurons and on broader neural circuits remain elusive. Our study reveals that *T. gondii* infection disrupts cytoskeletal dynamics in SH-SY5Y neuronal cells and primary cortical neurons. Infected neuronal cells undergo significant cytomorphological changes, including retraction of dendritic extensions and alterations in microtubule and F-actin networks, across both parasite genotypes I and II. These cytoskeletal alterations were notably diminished in cells exposed to *T. gondii* mutants with impaired secretion via the MYR translocon, and were independent of intraneuronal parasite replication. Moreover, a bystander effect was observed, with supernatants from *T. gondii*-challenged cells inducing similar cytoskeletal changes in uninfected cells. Analyses of extracellular vesicles (EVs) in supernatants revealed differential expression of host microRNAs in response to infection, most notably the upregulation of miR-221-3p, a microRNA not previously associated with *T. gondii*. The data indicate that unidentified parasite-derived effector(s) secreted via the MYR translocon, in conjunction with MYR-independently induced EV-associated host microRNAs, mediate cytoskeletal alterations in both infected and bystander neuronal cells. The findings provide new insights into molecular mechanisms by which *T. gondii* infection may disrupt neural networks, shedding light on its potential role in neuronal dysregulation.

**Supplementary Information:**

The online version contains supplementary material available at 10.1038/s41598-025-96298-8.

## Introduction

In the vertebrate central nervous system (CNS), essential neural functions are safeguarded by restrictive cellular barriers and immune surveillance^1^. However, a limited number of pathogens can establish infection in the CNS^2^. One such pathogen is *Toxoplasma gondii*, which invades and establishes chronic, latent infections in neurons^3^.

It is estimated that one-third of the global human population encounters *T. gondii* at some point in their lives^4^. Following oral or congenital infection, *T. gondii* disseminates widely throughout the body. Although infections are primarily asymptomatic in healthy individuals, they can lead to life-threatening encephalitis in immunocompromised persons, severe neurological complications in developing fetuses, and relapsing ocular manifestations in otherwise healthy individuals^5,6^. Numerous epidemiological studies have linked *T. gondii* carriage or seropositivity to an elevated risk of psychiatric disease, for example schizophrenia and neurocognitive impairment^7,8^.

The tissue-invasive fast-replicating stage of *T. gondii*, known as the tachyzoite, is obligate intracellular. Thus, the invasion of host cells, including neurons, is essential for its survival^9^. The active invasion of host cells is propelled by the parasite’s own actin-myosin motor^10^ and involves the discharge of secretory organelles into the host cell cytosol^11,12^. A secretory machinery, known as the MYR complex, ensures the transport of effectors across the intracellular parasitophorous vacuole where the parasite resides^13^. From this intracellular replicative niche, the secreted proteins modulate various host cell functions, including host cell signaling and gene expression^12^. Following systemic dissemination in the bloodstream, tachyzoites cross the blood–brain barrier and invade neurons, where they later persist chronically as microscopic bradyzoite cysts^6^.

Although astrocytes, microglia and neurons can readily become infected in vitro^14^, a preference for parasite localization to neurons has been identified^15–17^. While the molecular mechanisms underlying this preference remain largely unexplored, mounting evidence indicates that *T. gondii* infection induces alterations in neuronal functions (reviewed in^18^). Additionally, the mechanisms that allow chronic parasite persistence in neurons remain enigmatic^19^. Furthermore, studies in mice have shown altered neurotransmission following *T. gondii* infection^20–22^.

Mounting evidence shows that neurons secrete exosomes and other extracellular vesicles (EVs), which play important roles in inter-neuronal and intercellular communication within the CNS^23^. MicroRNAs (miRNAs), which may be packaged within these EVs, also influence neuronal communication by regulating the synthesis of proteins involved in synaptic transmission and other forms of neuronal signaling^24^. Therefore, miRNAs are crucial for normal CNS function, and their dysregulation has been associated with various diseases, including *T. gondii* infection^25^.

The ability of *T. gondii* to productively infect neurons during acute primary infection and then persist within neurons, thereby impacting neuronal cell function, is a key factor in the pathogenesis of toxoplasmosis. In this study, we utilized cellular models to investigate the potential mechanisms underlying the effects of *T. gondii* infection on the cytomorphology of neuronal cells.

## Results

### *T. gondii* infection induces morphological alterations in SH-SY5Y cells and primary cortical neurons

**Fig. 1:**
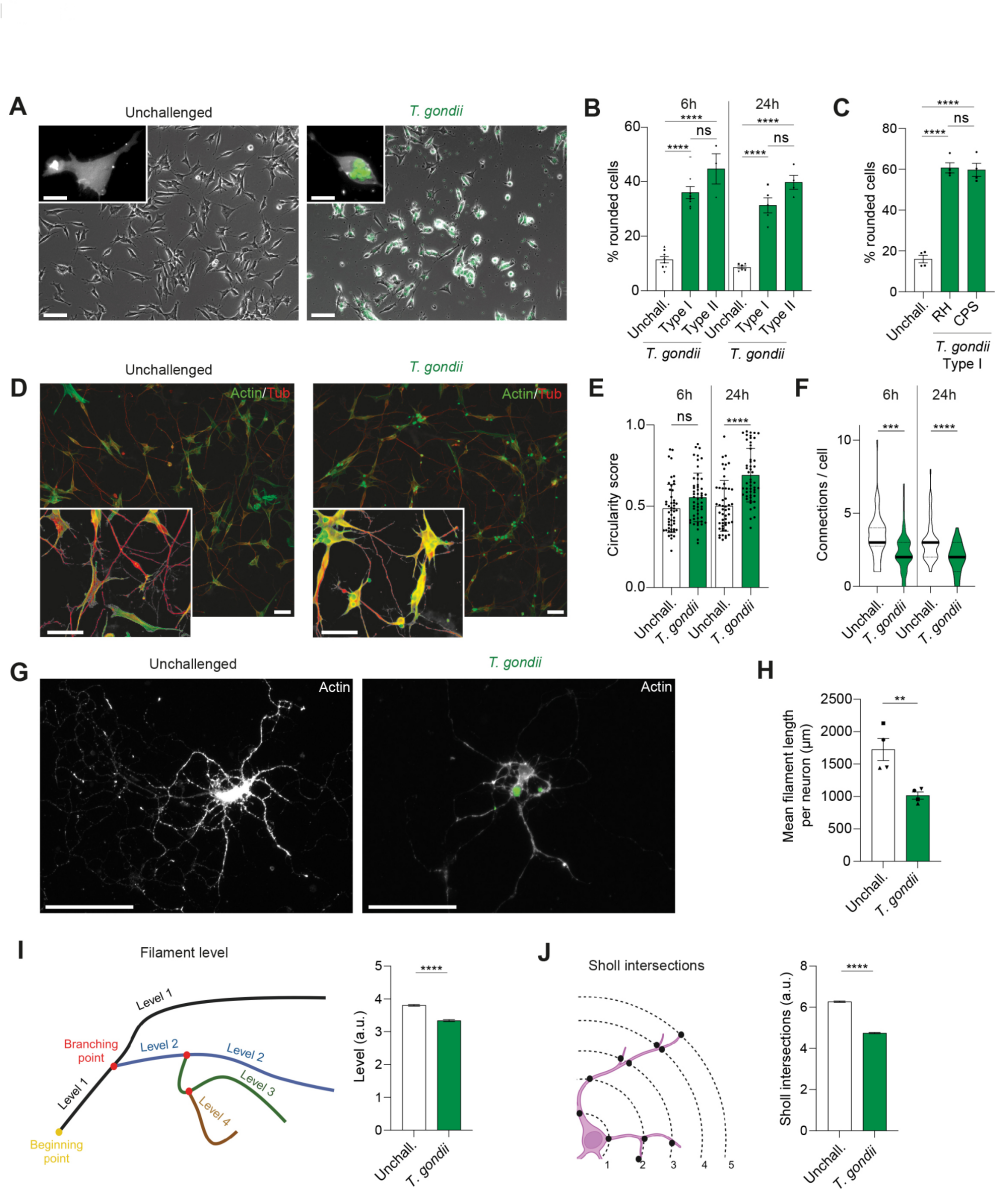
Impact of *T. gondii* infection on neuronal cytomorphology. SH-SY5Y cells and primary cortical neurons were challenged with indicated lines of freshly egressed *T. gondii* tachyzoites (MOI 1) or maintained in complete medium (unchallenged) for 6 or 24 h. (**A**) Representative micrographs of undifferentiated SH-SY5Y cells, unchallenged or challenged with GFP-expressing *T. gondii* (RH-LDM) for 24 h. Scale bars:100 µm, inset scale bars: 25 µm. (**B**, **C**) Percentage of cells (mean ± SEM) with rounded cell morphology related to the total cell count. SH-SY5Y cells were challenged with (**B**, **C**) RH-LDM (type I), (**B**) Pru-A7 (type II) and (**C**) RH-CPS (type I) for 6–24 h. Each data point represents one biological replicate with 100–200 cells counted/data point. Data from 3 to 8 independent experiments (n = 3–8). (**D**) Representative micrographs of differentiated SH-SY5Y cells unchallenged or challenged with GFP-expressing *T. gondii* (RH-LDM), stained for actin (green) and tubulin (red). Scale bars: 50 µm, inset scale bars: 50 µm. (**E**) Mean cell body circularity (± SEM) scored for differentiated SH-SY5Ycells, unchallenged or challenged with GFP-expressing *T. gondii* (RH-LDM) for 6–24 h. Each data point represents one cell body with 50–60 cells counted/condition, from 3 independent experiments (n = 3). (**F**) Violin plot of number of connections between differentiated SH-SH5Y cells treated as in (**E**) Dotted lines represent quartiles and bold lines medians. 50–100 cells were counted/condition, from 3 independent experiments (n = 3). (**G**) Representative micrographs of unchallenged and *T. gondii* (RH-LDM)-challenged cortical primary mouse neurons. Scale bar: 100 µm. (**H**–**J**) Primary neurons were challenged by *T. gondii* (RH-LDM) for 24 h. Cartoons show mean filament level (**H**) and Sholl intersections (**J**), respectively, analysed using software as detailed in Methods. Bar graphs show mean (± SEM) filament length (**H**), filament level (**I**) and Sholl intersections (**J**) for unchallenged and challenged neurons. a.u.: arbitrary units. A total of 64–88 neurons were analysed per condition, from 4 separate purifications of neurons (n = 4), with a range of 6.000–9.000 filaments and 13.000–15.000 Scholl intersections analysed per condition. Statistical analyses were performed with (**B**, **C**, **E**, **F**) ANOVA, (H) Student’s *t-*test, (**I**, **J**) Kruskal–Wallis, ** *P* < 0.005, *** *P* < 0.0005, **** *P* < 0.0001, ns: non-significant.

To determine if alterations in cell morphology arise upon *T. gondii* infection, we first employed SH-SY5Y cells, a human neuroblastoma-derived cell line broadly used in in vitro models to study neuronal function and differentiation^26^. SH-SY5Y cells were challenged with freshly egressed GFP-expressing tachyzoites (Fig. 1A) and morphology was microscopically assessed. Interestingly, at different time-points (6–24 h), infected SH-SY5Y were characterized by a significantly higher frequency of rounded cells, with loss of cell membrane protrusions, and similarly upon challenge with prototypic type I (RH) and type II (PRU) *T. gondii* lines (Fig. 1B, Figs. S1A, B). Equivalent morphological alterations took place in infected cells upon challenge with the invasive but non-replicative (uracil-auxotroph) line RH-CPS (Fig. 1C, Fig. S1C), indicating that replication or parasite vacuole growth was not mediating the changes. Video-microscopy revealed that morphological changes took place within 1 h post-challenge and were maintained throughout infection (Movie S1). Next, differentiated SH-SY5Y cells were challenged, stained for tubulin and actin (Fig. 1D) and assessed with automatized image analysis software. Interestingly, *T. gondii*-challenged cells presented an elevated circularity score (Fig. 1E) with reduced numbers of intercellular connections (Fig. 1F) and membrane protrusions (Fig. S1D). Finally, primary cortical neurons were challenged with *T. gondii* (Fig. 1G). Importantly, analysis software detected a reduction of the total major cellular ramification length in infected neurons (Fig. 1H), their ramification level (Fig. 1I) and the numbers of Sholl intersections (Fig. 1J). To determine whether cells were under considerable stress upon *T. gondii* challenge, we assessed the transcription of a number of genes linked to neurotransmission and cellular homeostasis. Transcription of this set of genes appeared unaffected in *T. gondii*-challenged primary neurons (Fig. S1E). The finding that *T. gondii* infection consistently evoked measurable cell morphological changes in parasitized neuronal cells and primary neurons motivated a further detailed analysis.

### Impact of intracellular parasite-derived secretions and host cell-derived secretions on the morphological alterations of SH-SY5Y cells

**Fig. 2:**
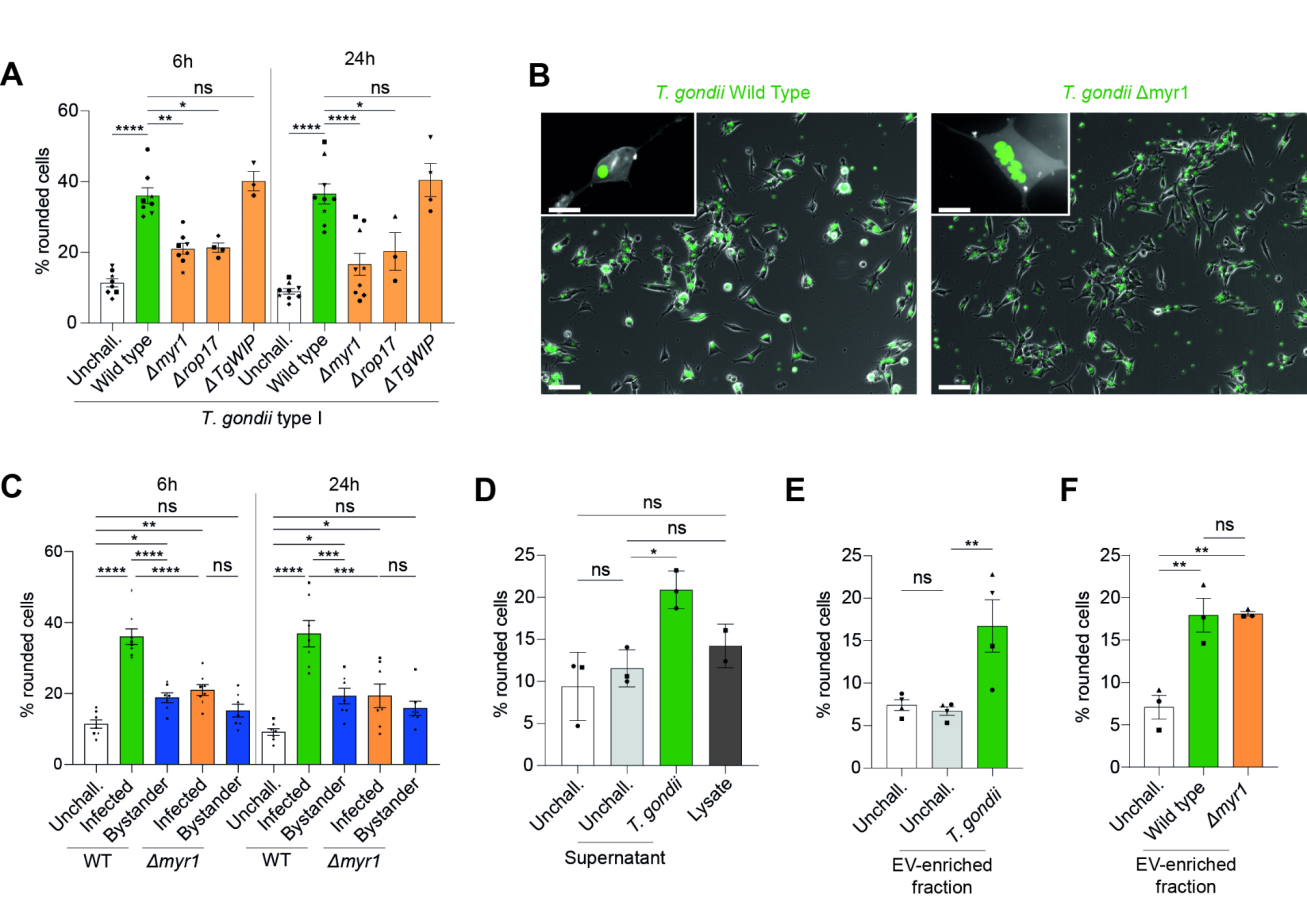
Impact of *T. gondii* secretory machinery on infected neuronal cell morphology and bystander effect on neighboring cells. (**A**) Percentage of cells (mean ± SEM) with rounded cell morphology related to the total cell count. SH-SY5Y cells were challenged with *T. gondii* wild type (RH, parental), *Δmyr1*, *Δrop17* and *ΔTgWIP* for 6–24 h from 3 to 8 independent experiments (n = 3–8). (**B**) Representative micrographs of SH-SY5Y cells challenged with GFP-expressing wild type (RH) and *Δmyr1* for 24 h. Scale bar:100 µm, inset scale bar: 25 µm. (**C**–**F**) Percentage of cells (mean ± SEM) with rounded cell morphology related to the total cell count. (**C**) SH-SY5Y cells were challenged with GFP-expressing *T. gondii* wild type (RH) and *Δmyr1,* MOI1 for 6–24 h*.* Infected cells were defined as GFP^+^ cells with replicating vacuoles and GFP^−^ cells considered bystander cells (n = 7–8). (**D**) Naïve SH-SY5Y cells were challenged with supernatant from *T. gondii*-challenged and unchallenged SH-SY5Y cultures at 1:1 (vol/vol) or *T. gondii* lysate (MOI-equivalent 2; n = 2–3). (**E**) Naïve SH-SY5Y cells were challenged with EV fractions (1:1, vol/vol) collected from unchallenged or *T. gondii* (RH, MOI1)-challenged SH-SY5Y cells (n = 4). (**F**) Naïve SH-SY5Y cells were challenged with EV fractions (1:1, vol/vol) collected from unchallenged or *T. gondii* wild type (RH)- or *Δmyr1-*challenged SH-SY5Y cells (n = 3). Each data point represents 100–300 counted cells from the indicated number of independent experiments (n). Statistical analyses were performed with ANOVA, * *P* < 0.05, ** *P* < 0.005, *** *P* < 0.0005, **** *P* < 0.0001, ns: non-significant.

To further evaluate the underlying cellular processes that lead to morphological changes, SH-SY5Y cells were challenged with parasite mutants that are defective in secreting effector molecules into the host cell (Δmyr1, Δrop17)^13^. Interestingly, challenge with either mutant similarly induced a significantly lower frequency of rounded cells (Fig. 2A), with maintained angularity and protrusions in infected cells (Fig. 2B, Fig S2A), compared with wild-type (WT) *T. gondii*. Next, we tested the effector *T.* *gondii* WAVE complex interacting protein (TgWIP) because of its implications in cytoskeletal podosome dissolution in immune cells^27,28^. Surprisingly, the TgWIP mutant (ΔTgWIP) and WT exhibited similar phenotypes (Fig. 2A), indicating differential effects in the cytoskeletal remodeling of phagocytes and neuronal cells. Further, we tested a panel of parasite mutants (Δgra15, Δgra24, Δgra28, Δrop16, ΔTgIST) deficient in effectors implicated in central cell signaling pathways^12^. However, these also presented phenotypes similar to WT (Fig. S2B, C). Next, in *T. gondii*-challenged cell cultures, we assessed the morphology of non-infected SH-SY5Y—bystander—cells and found significant morphological changes at a frequency similar to cells exposed to Δmyr1 and Δrop17 mutants (Fig. 2C), indicating a bystander effect. To confirm this, SH-SY5Y cells were exposed to supernatants from *T. gondii* WT-challenged SH-SY5Y cells. Supernatants from *T. gondii*-exposed SH-SY5Y cells, but not supernatants from unchallenged SH-SY5Y cells or *T. gondii* lysate, induced significant changes in morphology (Fig. 2D). Finally, SH-SY5Y cells were exposed to supernatant fractions enriched in EVs. Interestingly, EV fractions from *T. gondii*-challenged, but not unchallenged, SH-SY5Y cells induced measurable morphological alterations (Fig. 2E). In contrast, EVs purified from WT- and Δmyr1-challenged SH-SY5Y cells similarly induced alterations (Fig. 2F), indicative of a MYR-independent mechanism. Altogether, the data indicates that (1) MYR-dependent parasite-derived secretions into the infected SH-SY5Y cell impact cytomorphology and, that, (2) EV-enriched fractions secreted by cells upon *T. gondii* challenge also mediate morphological changes MYR-independently, in line with the observed bystander effect.

### Characterization of EVs and EV-mediated morphological alterations of SH-SY5Y cells

**Fig. 3:**
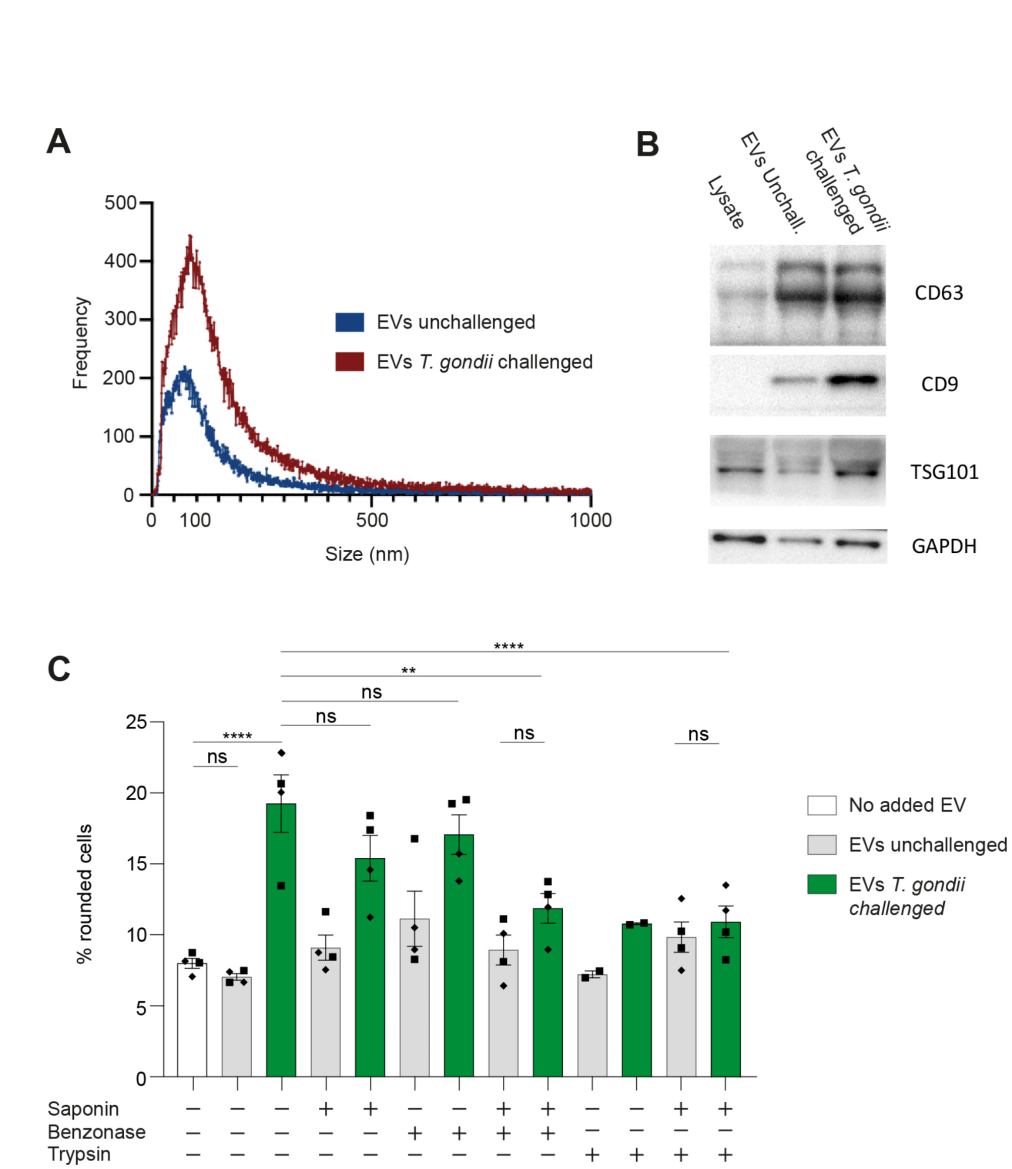
Characterizations of EVs secreted by *T. gondii*-challenged SH-SHY5Y cells and impact on cell morphology. (**A**) Nanoparticle tracking analysis (NTA) plot shows relative particle frequency and distribution of particle size of EV fractions collected from unchallenged and *T. gondii* (RH)-challenged SH-SY5Y cells. Representative of 2 independent experiments performed in triplicate. (**B**) Western blotting of lysates from EV fractions collected from unchallenged and *T. gondii* (RH)-challenged SH-SY5Y cells, immunoblotted for EV markers CD63, CD9 and TSG101. Lysates from naïve SH-SY5Y served as control and GAPDH as loading reference. Representative of more than 3 independent experiments. Original blots are presented in Fig. S3. (**C**) Percentage of cells (mean ± SEM) with rounded cell morphology related to the total cell count. Naïve SH-SY5Y cells were challenged with EV fractions (1:1, vol/vol) pretreated with saponin, benzonase and trypsin, as indicated. Each data point represents 100–300 counted cells from 2 independent experiments performed in duplicate. Statistical analyses were performed with ANOVA, ** *P* < 0.005, *** *P* < 0.0005, **** *P* < 0.0001, ns: non-significant.

To determine the abundance and size of EVs secreted by SH-SY5Y cells upon challenge with *T. gondii*, EV-enriched fractions from cell supernatants were first subjected to nanoparticle tracking analysis (NTA)^29^. The highest relative abundance of detected nano- and microparticles had a diameter of 50–150 nm, peaking at ~ 100 nm (Fig. 3A, Movie S2). Interestingly, the relative abundance of these particles was increased in fractions from *T. gondii*-challenged cells. Further, Western blotting analyses revealed elevated expression of typical EV membrane glyco/protein markers (CD63, CD9, TSG101) in EV fractions (Fig. 3B, Fig. S3), consistent with enrichment for EVs^30^. Finally, to approach the molecular origin of the morphological effects observed in cells, EV fractions were subjected to nuclease and protease treatments in presence or absence of the membrane permeabilizing agent saponin. Treated EV fractions induced morphological changes at lower frequency in SH-SY5Y cells (Fig. 3C), indicating roles played by EV-associated nucleic acids and polypeptides in the phenotype. This motivated a further exploration of nucleic acids in EVs.

### Identification of EV-associated miRNAs with an impact on SH-SY5Y cell morphology

**Fig. 4:**
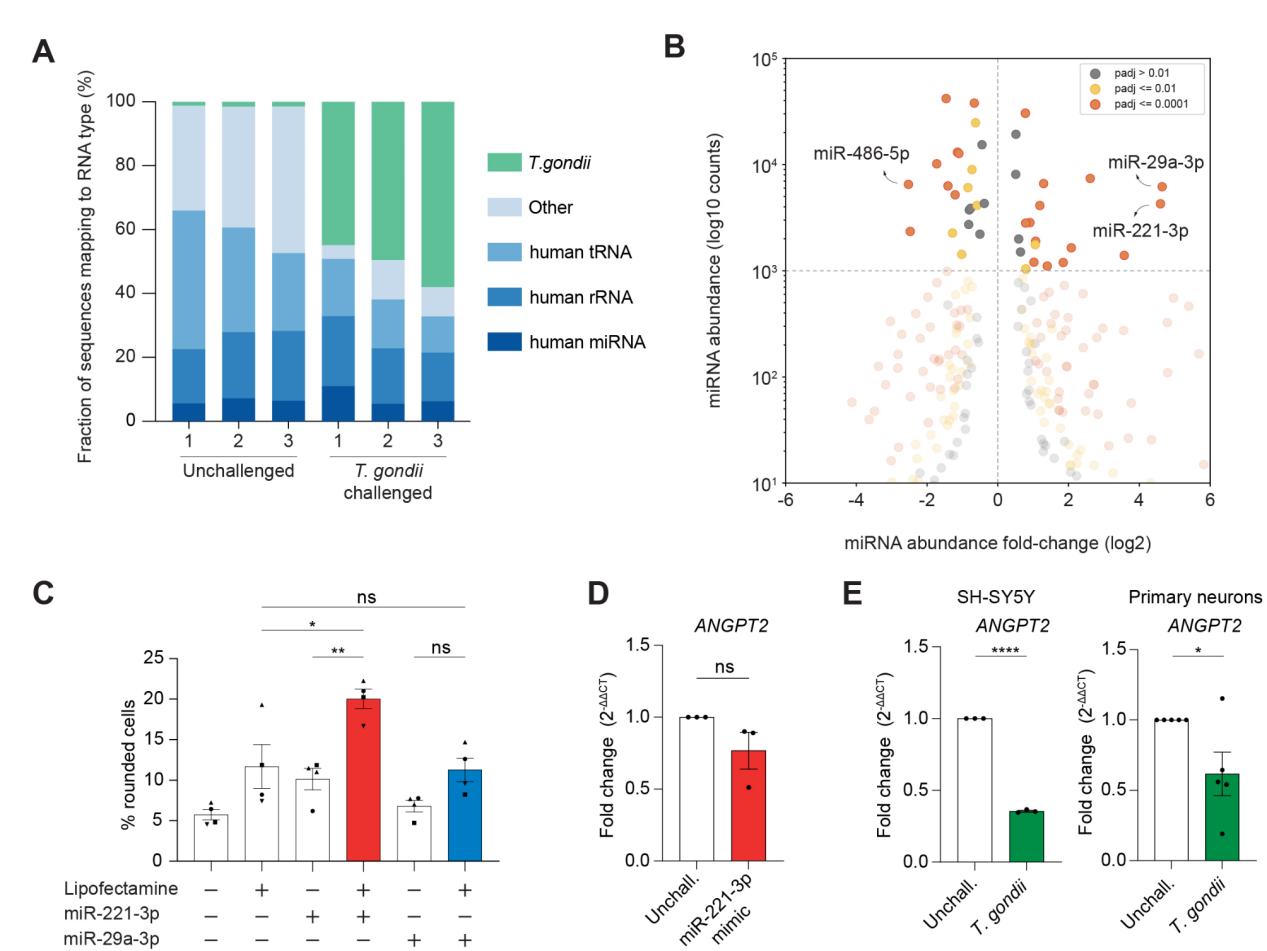
Characterizations of EV-associated miRNAs upon *T. gondii* challenge and impact on neuronal cell morphology. (**A**) small RNAs in EVs purified from supernatants of unchallenged or *T. gondii* (RH)-challenged SH-SY5Y cells. The Fraction (%) shows the percentage of each RNA type. Each sample (1–3) represents one independent biological replicate (n = 3). (**B**) Volcano plot of miRNA abundances in EV-enriched fractions from *T. gondii*-challenged vs. unchallenged cells. The vertical axis shows normalized miRNA abundances as measured with RNA next-generation sequencing. The dotted horizontal line indicates miRNAs that are detected an average of 1000 times in each dataset. The horizontal axis indicates the abundance fold-change of each miRNA. The vertical dotted line indicates no change in abundance upon the *T. gondii* challenge. (**C**) Percentage of cells (mean ± SEM) with rounded cell morphology related to the total cell count. Naïve SH-SY5Y cells were transfected with miR-221-3p mimic or miR-29a-3p mimic using lipofectamine. Each data point represents 100–300 counted cells (n = 4). (**D**, **E**) qPCR analyses of *ANGPT2* cDNA from undifferentiated SH-SY5Y cells transfected with miR-221-3p mimic (**D**) or SH-SY5Y cells and primary cortical neurons challenged with *T. gondii* (RH) for 24 h (**E**). qPCR data are displayed as fold change (2^−ΔΔCt^) in relation to unchallenged condition (n = 3–5). (n) indicates number of independent experiments. Statistical analyses were performed with (**C**) ANOVA, (**D**, **E**) Student’s *t-*test * *P* < 0.05, ** *P* < 0.005, *** *P* < 0.0005, ns: non-significant.

The prevalence of small RNAs such as microRNAs in EVs is well-established^31^. To assess the putative implication of these regulatory molecules, EV-enriched fractions were subjected to RNA next-generation sequencing and the relative abundance of small RNAs in each sample was measured (Fig. 4A). In the EVs from unchallenged cells, we found the presence of microRNAs (miRNAs) as well as sequences from transfer RNAs (tRNAs) and ribosomal RNAs (rRNAs). The presence of these molecules in small RNA fractions is common^32^. In the EVs from cells challenged with *T. gondii*, we additionally found the presence of sequences originating from the parasite genome (Fig. 4A). Since miRNAs are known to be potent regulators of gene expression and biological processes, we next studied the levels of various human miRNAs in the EVs from challenged and unchallenged cells. Our stringent analyses revealed 9 miRNAs that are down-regulated and 16 miRNAs that are up-regulated in EVs upon the challenge (Fig. 4B). We considered only miRNAs of substantial abundance (above the horizontal dotted line), since these are most likely to have regulatory effects^33^ (Tab. S1). Out of these, miR-29a-3p, miR-221-3p and miR-486-5p were chosen for further investigation because of their dramatic change in abundance in secreted EVs upon *T. gondii* challenge and their known associations with cell signaling and CNS pathologies^34–37^. Preliminary experiments suggested that miR-486-5p did not induce an increase in cell rounding and this candidate was not pursued further. Interestingly, transfection of SH-SY5Y cells with miR-221-3p induced morphological changes, while transfection with miR-29a-3p yielded non-significant differences compared with mock transfection (Fig. 4C). Next, we measured the effect of miR-221-3p on a reported target RNA in endothelial cells, angiopoietin 2 (*ANGPT2*)^38^, and measured a tendency for downregulation SH-SY5Y cells, without reaching statistical significance (Fig. 4D). However, the expression of *ANGPT2* was downmodulated in both SH-SY5Y cells and primary neurons upon *T. gondii* challenge (Fig. 4E). Jointly, the data indicate that *T. gondii* infection modulates the expression of miRNAs and that elevated expression of miR-221-3p impacts the morphology of neuronal cells.

## Discussion

In this study, we investigated the impact of *T. gondii* infection on neuronal cell morphology. Our data show that, upon challenge with *T. gondii,* both a neuronal cell line and primary neurons undergo significant morphological changes, driven by (1) the intracellular presence of the parasite and (2) secreted factors from host cells.

We demonstrate that *T. gondii* infection induces cytoskeletal alterations in both undifferentiated and differentiated SH-SY5Y cells, as well as in primary neurons. The most prominent and easily quantifiable morphological change -cell rounding- was observed in undifferentiated SH-SY5Y cells. In differentiated cells, however, we additionally detected a reduction in the number of cell protrusions and connectivity. These findings are consistent with the more subtle changes observed in primary neurons, which retained major dendrites but showed diminished thin filament branches and Sholl intersections. The diverse, yet consistently observed, cytoskeletal alterations may indicate varying responsiveness to *T. gondii* infection across different neuronal cell types. This variation is likely influenced by factors such as differentiation state, adhesion, and connectivity^39^, and could be particularly relevant to infections in developing fetuses, upon creation of neural networks. Additionally, the implications of these cytoskeletal changes in neuronal cells likely differ from the motility- and migration-related changes observed in immune cells^28,40,41^. Hypothetically, these alterations in cell connectivity could affect neuronal function, potentially influencing synaptic connections and dendritic spine formation, though this assumption warrants further investigation. These findings also align with recent studies showing that *T. gondii* infection alters neuronal electrophysiology^42–45^ and neurotransmission in mice^20–22^.

We report that intracellular secretions through the MYR translocon^13^ mediate cytoskeletal alterations in neuronal cells. Specifically, two separate parasite mutants with impaired MYR-mediated transport across the parasitophorous vacuole membrane (∆myr1 and ∆rop17) similarly failed to induce cytoskeletal changes. In contrast, a non-replicating uracil-auxotroph mutant exhibited a phenotype similar to that of the wild-type. Together, these data indicate that secreted effectors associated with the intracellular localization of the parasite, rather than replication per se, mediate the cytoskeletal alterations. Unexpectedly, the effector TgWIP, which is known to mediate cytoskeletal alterations and podosome dissolution in phagocytes^27,28^, did not appear to play a critical role in the cytoskeletal changes of neuronal cells, suggesting differences in responsiveness between immune and neuronal cells. Furthermore, we screened parasite effector mutants linked to important signaling pathways in immune cells (NF-κB, p38 MAPK, STAT) and observed a phenotype similar to that of wild-type. This collectively corroborates the differences in responses to intracellular *T. gondii* between immune cells and neuronal cells, possibly linked to variations in cytoskeletal regulation or tighter regulation in sessile neuronal cells. We postulate that yet unidentified effector(s) secreted through the MYR translocon mediate the cytoskeletal alterations in neurons. Surprisingly, despite the impact of *T. gondii* on the GABAergic machinery in phagocytes^46–48^, we did not detect any effects on the GABAergic transcriptional machinery. This suggests stricter regulation in neurons or a lack of responsiveness in this neuronal subset. In contrast, the intracerebral delivery of EVs in mice has been reported to disrupt catecholaminergic signaling^49^. The intriguing possibility of EV-mediated paracrine effects on neurons in vivo warrants further investigation.

We demonstrate that EV-enriched fractions from *T. gondii*-challenged neuronal cells impact the cytoskeleton and overall morphology of non-infected neuronal cells. Initially, the consistent bystander effect indicated this possibility, which was confirmed using supernatants from infected cells. Additionally, the elevated total amounts of secreted EVs and the differential expression of miRNAs in EV-enriched fractions from *T. gondii*-challenged SH-SY5Y cells are noteworthy in this context. The reduction of effects following nuclease and protease treatments supports the notion of EV-mediated effects, although it cannot be fully excluded that additional factors, such as secreted cytokines, may also contribute. Taking into consideration the multiple and heterogeneous pathways described for EV uptake by cells and for effects inside cells^50^, the loss of phenotype upon nuclease treatment of EVs -only in presence of permeabilization- may be consistent with localization of miRNAs inside EVs. Further, the loss of phenotype upon protease treatment in absence of permeabilization may be consistent with a need of protein components for uptake of EVs and/or a direct implication of polypeptides in the phenotype. Regardless, purified EV fractions from *T. gondii*-challenged SH-SY5Y cells induced cytoskeletal alterations in naïve cells, and independently of MYR. Collectively, these data provide in vitro proof of concept that the effects induced by the parasite in infected neuronal cells could propagate to neighboring or even distantly located neurons. This also raises the intriguing question of how a relatively low number of *T. gondii*-infected neurons may elicit complex, long-lasting behavioral changes in rodents^51^. Our data align with models of chronic toxoplasmosis, and provide potential cellular and molecular mechanisms for the observed reductions of neuronal dendritic arborization and synaptic connectivity in *T. gondii*-infected rodents^43–45^.

We identify several upregulated and downregulated host miRNAs in the EV-enriched fractions of *T. gondii*-challenged neuronal cells. However, we also found substantial amounts of RNA sequences originating from the *T. gondii* genome in the EV-enriched fractions of the challenged neuronal cells. Given the incomplete state of the small RNA annotations of *T. gondii*, further investigations into these will require extensive de novo gene annotation efforts and will be the focus of future studies. Surprisingly, we also found minute traces of *T. gondii* sequences in EVs from the unchallenged samples. These sequences map to the parasite genome with high confidence and likely represent minute contaminations that can occur during sample preparation, next-generation sequencing library preparation, or even during sequencing as samples indices are misassigned by the instrument optics^52^. Importantly, such minute contaminations are unlikely to perturb our analyses or conclusions^32^.

We report that miR-221-3p can induce cytoskeletal changes in SH-SY5Y cells, similar to those of *T. gondii* infection. To our knowledge, miR-221-3p is a particularly interesting target, as it has not been previously associated with *T. gondii* infection but is linked to neuroprotection and nerve regeneration^53,54^. Other differentially expressed miRNAs with known associations to neuronal functions include miR-29a-3p and miR-486-5p; however, our data suggest that these miRNAs are not primarily driving cytoskeletal alterations in SH-SY5Y cells. Surprisingly, miR-155, which has been previously associated with *T. gondii* infection^25^, did not appear to be modulated in our analyses. This may indicate these miRNAs have a closer association with inflammatory and immune cell responses rather than direct neuronal responses. In line with this, it is noteworthy that the cytoskeletal phenotypes remained unchanged when challenged with parasite effector mutants linked to central immune signaling pathways. Given that NF-κB, MAPK, and STAT signaling play important roles in *T. gondii* infection and that miRNAs are known to target these pathways^55,56^, the maintained cytoskeletal changes upon challenge with GRA15, GRA24 or ROP16 mutants suggest that the cytoskeletal alterations described here are cell-specific to neurons and that separate responses occur in immune cells. Furthermore, this raises questions regarding the interactions between neurons, astrocytes, microglia and infiltrating leukocytes, which critically influence neuroinflammatory responses in the CNS. Whether *T. gondii*-infected neurons and their secretions contribute to neuroinflammation or its mitigation remains an open question. We envision that responses could be diverse, exhibiting both pro- and anti-inflammatory components, with reciprocal effects mediated by EVs/miRNAs, warranting further investigation. An enhanced understanding of the mechanisms by which *T. gondii* modulates neuronal function is critical for comprehending toxoplasmosis and for developing targeted approaches to limit its impact.

## Methods

### Ethical approval

The Regional Animal Research Ethical Board, Stockholm, Sweden, approved experimental procedures and protocols involving extraction of cells from mice (permit number 16403-2022), following proceedings described in EU legislation (Council Directive 2010/63/EU). All methods were carried out in accordance with relevant guidelines and regulations. All methods are reported in accordance with ARRIVE (Animal Research: Reporting of In Vivo Experiments) guidelines (https://arriveguidelines.org).

### Animals

Male and female C57BL/6NCrl mice (purchased from Charles River Laboratories, strain code 027) were bred at the Stockholm University Animal Facility. 1–2 day old mouse pups were sacrificed by decapitation. A total of 7 mice were used. Dams and pups were housed in a ventilated facility, provided with unrestricted access to tap water and food, and kept under a 12-h light/dark cycle at a temperature of 20–22 °C, with wood shavings as bedding material.

### Primary neurons

Cortices were sliced in 1 mm thin pieces before trypsin (0.25%, Sigma-Aldrich) treatment at 37 °C for 10 min. The cortex pieces were then triturated, resuspended and allowed to settle for 2 min before collecting supernatant. Neurons were collected by centrifugation at 200 g for 4 min and plated on Matrigel or Poly-L-Lysine (0.01%, Sigma-Aldrich) -coated glass coverslips and grown in Neurobasal media (ThermofischerScientific) supplemented by B27 (ThermofischerScientific), 0.5 mM L-Glutamine (HyClone) and gentamycin (20 µg/ml, Gibco). Primary neurons were used in experiments 5 to 12 days after isolation.

### SH-SY5Y cell culture and differentiation

The SH-SY5Y neuroblastoma cell line (CRL-2266, American Type Culture Collection) was cultured in OptiMEM (Gibco, Invitrogen) supplemented with 10% Fetal Bovine Serum (FBS; Cytiva, HyClone), defined as complete medium (CM). Differentiation of SH-SY5Y cells was induced by 1 μM retinoic acid in OptiMEM supplemented with 5% FBS. Medium was changed every 2-3 days and cells were used 7 days after induction. All cell cultures were grown in a humidified atmosphere containing 5% CO2 at 37°C.

### Parasites

*Toxoplasma gondii* tachyzoites were maintained by serial 2-day passages in human foreskin fibroblast (HFF-1 SCRC-1041, American Type Culture Collection) monolayers cultured in Dulbecco’s modified Eagle’s medium (DMEM, VWR) with 10% FBS, gentamicin (20 μg/ml, Gibco). Freshly egressed *T. gondii* tachyzoites of parental lines and mutants in Tab. S2 were used for experiments. *T gondii* lysate was obtained by 5 freeze–thaw cycles from 37 to −80 °C. All cell cultures used were periodically tested for mycoplasma and found to be negative.

### Immunocytochemistry and image analysis

Undifferentiated and differentiated SH-SY5Y cells were challenged and live imaged or imaged after fixation using Zeiss Observer Z1 or Leica DMi8 widefield microscopes. For each preparation, micrographs from 2–4 randomly chosen fields of view were acquired for assessment. An average of 100–200 cells per biological replicate were ocularly assessed for cell shape: elongated/angular *versus* rounded morphology (with absence of angular shapes).

Differentiated SH-SY5Y cells were incubated with mouse anti-β-tubulin polyclonal antibody (Invitrogen) ON at 4 °C followed by Alexa Fluor 594-conjugated secondary antibodies (ThermofisherScientific), Alexa Fluor 488-conjugated phalloidin (Invitrogen). Coverslips were mounted with DAPI and imaged. Image analysis was performed with FIJI Image J, version 2.0.0 for the measurements: (1) cell body circularity: *4π(area/perimeter*^2^*), where 0* = *non-circular and 1* = *circular and* (2) connections with other cells, both neurite-neurite and neurite-cell body.

Primary mouse cortical neurons were permeabilized using 0.3% Triton X-100 (Sigma-Aldrich) in PBS, incubated with Phalloidin-AlexaFluor594 (Invitrogen) for 1.5 h before glass coverslips were mounted on slides and imaged using a Leica DMi8 widefield microscope. Image analysis was done using Imaris v10.1 sofware and neuronal network complexity was assessed using functions: Filament Number of Sholl Intersections with a 1.0 μm step resolution for the spheres; Dendrite Branch Level and Filament Length. Filament Level is a numerical structure that starts unfolding from the filament beginning toward the terminal points, assigning Branch Level to dendrite segments at each branching point. Sholl analysis creates a series of concentric circles (spheres in 3D) around the beginning point of the filament. Number of Sholl Intersections is defined as the number of dendrite intersections (branches) on concentric spheres, defining dendrite spatial distribution as a function of distance from the beginning point.

### Isolation of EVs, Nanoparticle tracking analyses (NTA) and treatments of EV fractions

SH-SY5Y cells were grown in T175 culture flasks in EV-free CM and challenged with freshly egressed *T. gondii* at MOI 2 for 24 h to reach 80–90% infection frequency. Supernatants were collected, centrifuged at 2000 g for 45 min at 4 °C to remove cell debris. Supernatants were then either used in experiments or spun at 100 000 g (Beckman Coulter L-90 K ultracentrifuge) for 1 h at 4 °C to collect EV-enriched fractions. Fractions were then washed with PBS before another round of centrifugation at 100 000 g,1 h at 4 °C. Fractions were kept in PBS and stored at − 80 °C until use.

NTA was performed on a NanoSight Pro (Malvern Panalytical). Briefly, EV-enriched fractions were diluted in PBS (1:2), placed into sample chamber and the particles sizes and numbers were recorded for 90 s.

For enzymatic treatments, freshly collected EV fractions were permeabilised with 0.01% saponin (Sigma), followed by benzonase (100 U/ml; Merck) treatment for 10 min at RT or trypsin (50 µg/ml; Sigma) treatment at 37 °C for 15 min. Benzonase was stopped by adding 20 mM EDTA and trypsin by adding CM. Treated EV fractions were subsequently used to challenge naïve SH-SY5Y.

### Western blotting

Cells and EV-enriched fractions were lysed with RIPA buffer (150 mM NaCl, 0.1% Triton X- 100, 0.5% sodium deoxycholate, 0.1% sodium dodecyl sulphate (SDS), 50 mM Tris- HCl, pH 8.0, protease inhibitors and phosphatase inhibitors) on ice, followed by homogenization by passing 10 times through an 27 g needle. Samples were then centrifuged for 20 min at 12000 g at 4 °C and the supernatant aspirated and aliquoted. Laemmli gel loading buffer (Bio- Rad) was added to each sample and boiled at 95 °C for 5 min, after which proteins were separated on 10% SDS-PAGE gel, and blotted onto PVDF membranes (Millipore), blocked in 5% BSA followed by Western blotting with primary antibodies: GAPDH (Millipore), CD63, CD9 (ExoAB Antibody kit, SBI System Biosciences) and TSG101 (Invitrogen). Proteins were revealed by enhanced chemiluminescence (GE Healthcare) in a BioRad ChemiDocTM system (BioRad).

### Small RNA-seq library preparation and sequencing of RNA extracted from EVs

Small RNA sequencing libraries were prepared using the QIAseq miRNA Library Kit (Protocol handbook July 2020) with some customization. 5 µl of the prepared EV samples were used as input. The 3’ and 5’ adapters used in the adapter ligation reactions, as well as the reverse transcription primers were diluted as follows: 3´ adapter: non-infected samples 1:5, infected samples 1:10; 5’ adapter: non-infected samples 1:5, infected samples 1:2.5; reverse transcription primers: non-infected samples 1:10, infected samples 1:5. After reverse transcription the samples were amplified for 22 (non-infected samples) or 20 (infected samples) PCR cycles according to the QIAseq protocol (15 min at 95 °C, 20 × or 22x (15 s at 95 °C, 30 s at 60 °C, 15 s at 72 °C), 2 min at 72 °C, hold at 4 °C) with sample specific index primers following the protocol section “Library Amplification Using HT Plate Indices”. The PCR reaction was purified using the kit provided reagents, but omitting the upper size selection to retain larger fragments. As such, 37.5 µl of QIAseq miRNA NGS Bead Binding Buffer without beads was added to the amplified libraries and the samples incubated at RT for 10 min. Then the samples were mixed with 65 µl of QMN beads and incubated for 10 min at RT. After ethanol washes and air drying of the beads, elution of the libraries was performed in 17 µl of nuclease free water for 1 h at RT and the libraries stored at -20 °C until sequencing. Samples were sequenced on NextSeq2000 (NextSeq 1000/2000 Control Software 1.4.1.39716/RTA 3.9.25) with a 101nt(Read1)-8nt(Index1)-8nt(Index2) setup using a ‘P2’ flowcell. The Bcl to FastQ conversion was performed using bcl2fastq_v2.20.0.422 from the CASAVA software suite. The quality scale used is Sanger / phred33 / Illumina 1.8+.

### Read quality control and mapping

To correct for PCR duplicates, we developed a custom script to perform UMI correction on the raw reads, retaining only the unique combinations of [sequence + UMI] without altering the overall read structure inherent to the QIAseq sequencing method ([small RNA sequence + 3' adapter + UMI]) at a maximum length of 50 nucleotides. The sequence quality control and adapter trimming of the UMI-corrected reads were performed using the miRTrace tool (version 1.0.0)^32^ as “mirtrace qc -s hsa -a AACTGTAGGCACCATCAAT -w *.fastq.gz -o output_folder”. The quantification of human miRNA reads was performed using ‘quantifier’ from miRDeep2 (version 2.0) as “*quantifier.pl -p hsa_hairpin_miRBase.fa -m hsa_mature_miRBase.fa -r reads_UMI_corrected.fa -t hsa*”. To calculate the proportion of the unknown reads that map to the *T. gondii* genome we stringently mapped the *unknown*-labelled reads from the miRTrace report against the parasite genome (Toxoplasma_gondii.TGA4.dna.toplevel.fa) using bowtie tool (version 1.0.0) as “*bowtie -v 0 tg -a –best –strata -f reads_UMI_corrected_unknown.fa*”.

### Differential gene expression analysis

DESeq2 package (version 1.36) of R was used for performing differential gene expression analysis. Differentially expressed genes were identified by adjusted p-value of less than 0.05. Benjamini–Hochberg method was applied for the p-value adjustment and the contrast was set to the infected vs non-infected samples.

### Gene ontology analysis

All targets for differentially expressed miRNAs were obtained from TargetScanHuman (Predicted targets for conserved miRNA families) database. The top 100 target genes of differentially expressed miRNAs were selected based on having the weighted context++ score sorted from lowest to highest. GO analysis was performed using the R package Clusterprofiler (version 4.4). To select GO terms that pass a significant cutoff (less than 0.05), *p*-values were adjusted using the Benjamini–Hochberg method. The org.Hs.eg.db database was used as the annotation source for human gene ontology and all genes listed in TargetScan (hsa) were used as the background set.

### Transfections

Transfections were performed following the Lipofectamine 2000 protocol (ThermofischerScientific). Briefly, SHSY5Y cells were plated (24-well plates) the day before experiment to achieve 70% confluency. miRNA mimics mir-221-3p, mir-29a-3p and mir-486-5p (ThermofischerScientific) were selected and mir-mimics-Lipofectamine complexes were prepared as followed: 10 pmol miR-mimic in 50 µl serum-free OptiMEM, 0.5 µl Lipofectamine 2000 (ThermofischerScientific) in 50 µl serum-free OptiMEM. After 5 min at RT, solutions were combined and left standing for another 15 min at RT before 50 µl of transfection complex was added to each well.

### Quantitative polymerase chain reaction (qPCR)

Cortical neurons or SH-SY5Y cells, challenged as indicated, were lysed in Lysis buffer (Jena Bioscience), total RNA was extracted using the Total RNA Purification kit (Jena Bioscience) and reverse transcribed with Maxima H Minus Reverse Transcriptase (Thermo Fisher). Real-time qPCR was performed with SYBR® green PCR master mix (KAPA biosystems) specific forward and reverse primers at target-dependent concentrations (100 nM), and cDNA (10 ng) in a QuantStudio 5 System (Thermo Fisher) with ROX as a passive reference. qPCR results were analysed using the ΔCt method relative to Importin-8, TATA-binding protein (TBP) and glyceraldehyde-3-phosphate dehydrogenase (GAPDH) as housekeeping gene and displayed as fold change to unchallenged (set to 1). Primer sequences are listed in the Tab. S1.

### Statistical analyses

All statistical analyses were performed with Prism software (v.8, GraphPad). *p*-values < 0.05 were defined as significant.

## Electronic supplementary material

Below is the link to the electronic supplementary material.

Supplementary Material 1

Supplementary Material 2

Supplementary Material 3

Supplementary Material 4

## Data Availability

The datasets generated and analysed during the current study are available in the Sequence Read Archive (SRA) repository with the primary accession code PRJNA1193507.
