# Supplementary material for: Cytoskeletal alterations in neuronal cells implicate Toxoplasma gondii secretory machinery and host microRNA-containing extracellular vesicles
Source: Sci Rep. 2025 Apr 12;15:12606. doi: 10.1038/s41598-025-96298-8 (PMC11993698; doi:10.1038/s41598-025-96298-8)
Supplement: Supplementary file 3 — Supplementary Material 3 [file 41598_2025_96298_MOESM3_ESM.pdf]

Supplementary material file

Cytoskeletal alterations in neuronal cells implicate *Toxoplasma gondii* secretory machinery and host microRNA-containing extracellular vesicles

Thomas Mazza[1], Morteza Aslanzadeh[2], Lïse Berentsen[1], Franziska Bonath[3], Marc R. Friedländer[2], Antonio Barragan[1,*]

[1]Department of Molecular Biosciences, The Wenner-Gren Institute, Stockholm University, Stockholm, Sweden
[2]Science for Life Laboratory, Department of Molecular Biosciences, The Wenner-Gren Institute, Stockholm University, Stockholm, Sweden
[3]Kungliga Tekniska Högskolan, School of Engineering Sciences in Chemistry, Biotechnology and Health at the Department of Gene Technology, Stockholm, Sweden

Supplementary Table S1
Supplementary Table S2
Supplementary Figure S1
Supplementary Figure S2
Supplementary Figure S3

Supplementary Table S1: Selected miRNAs. baseMean and pvalue/pvalue adjusted

miRNAs	baseMean	log2FoldChange	pvalue	padj
**hsa-miR-29a-3p**	**6188.92358**	**4.64322**	**3.26E-120**	**2.49E-117**
**hsa-miR-221-3p**	**4273.05668**	**4.59761**	**1.39E-41**	**2.66E-39**
hsa-miR-100-5p	1394.40391	3.57277	7.08E-33	6.01E-31
hsa-miR-21-5p	7404.69117	2.61097	4.81E-39	7.34E-37
**hsa-miR-486-5p**	**6507.08980**	**-2.52950**	**5.42E-15**	**1.88E-13**
hsa-miR-1307-3p	2345.87481	-2.47861	1.18E-09	1.83E-08
hsa-miR-199b-5p	1645.27024	2.07560	8.55E-19	4.08E-17
hsa-miR-19b-3p	1195.22527	1.84718	2.03E-08	2.67E-07
hsa-miR-125a-5p	10173.55697	-1.72875	8.31E-09	1.11E-07
hsa-miR-92a-3p	41905.19553	-1.46330	2.15E-10	3.73E-09
hsa-miR-342-3p	6297.30157	-1.41191	6.26E-09	8.69E-08
hsa-miR-127-3p	1107.21585	1.39651	3.26E-08	4.08E-07
hsa-miR-143-3p	6625.61399	1.29301	3.13E-10	5.08E-09
hsa-miR-423-5p	2264.99073	-1.28340	7.61E-05	4.84E-04
hsa-miR-99b-5p	5194.22646	-1.20728	4.49E-08	5.52E-07
hsa-miR-27b-3p	4114.60500	1.18456	5.71E-07	6.14E-06
hsa-miR-122-5p	13067.63325	-1.14272	2.41E-07	2.74E-06
hsa-miR-191-5p	12712.82981	-1.10212	7.36E-07	7.59E-06
hsa-miR-30a-5p	1894.42860	1.06694	6.25E-09	8.69E-08
hsa-miR-148a-3p	1760.46369	1.05688	1.10E-04	6.77E-04
hsa-miR-423-3p	1426.70154	-1.02223	7.46E-04	3.63E-03
hsa-miR-152-3p	1203.64612	1.01955	1.90E-06	1.81E-05
hsa-miR-23a-3p	2842.33721	0.90733	4.58E-06	3.80E-05
hsa-miR-320a-3p	6061.34345	-0.84602	1.82E-03	8.01E-03
hsa-miR-320d	2726.18063	-0.82377	4.93E-03	1.86E-02
hsa-miR-320c	3737.79999	-0.80976	4.72E-03	1.80E-02
hsa-miR-148b-3p	1017.08267	0.80059	2.60E-03	1.10E-02
hsa-miR-186-5p	1042.49061	0.78854	1.99E-05	1.41E-04
hsa-miR-24-3p	2811.93834	0.78561	1.74E-06	1.68E-05
hsa-miR-199b-3p	30455.86448	0.77751	2.45E-06	2.23E-05
hsa-miR-199a-3p	30455.80851	0.77751	2.45E-06	2.23E-05
hsa-miR-320b	3878.57933	-0.77050	6.20E-03	2.28E-02
hsa-miR-30d-5p	8967.92677	-0.73115	3.96E-05	2.68E-04
hsa-miR-93-5p	37930.46767	-0.66670	3.74E-06	3.17E-05
hsa-miR-34a-5p	1502.85070	0.64072	1.14E-02	3.80E-02
hsa-miR-25-3p	24747.98572	-0.63163	1.50E-05	1.11E-04
hsa-let-7g-5p	1991.63450	0.58779	2.54E-03	1.09E-02
hsa-miR-425-5p	4131.96287	-0.58644	1.78E-03	7.89E-03
hsa-let-7i-5p	19284.85683	0.51002	2.47E-03	1.07E-02
hsa-miR-361-5p	2212.10115	-0.50806	4.12E-03	1.61E-02
hsa-miR-26a-5p	8088.52306	0.50514	4.65E-03	1.78E-02
hsa-miR-103a-3p	15373.95400	-0.44938	4.52E-03	1.74E-02
hsa-miR-151a-3p	4307.51706	-0.38364	1.08E-02	3.62E-02

Selected miRNAs for experiments are shown in bold character

Supplementary Table S2: Parasite lines, mutants and qPCR primers used in the study

T. gondii lines and mutants	References
T. gondii RH-LDM GFPS65T	[1,2]
T. gondii RH1-1 cLuc GFP⁺	[3]
T. gondii RH Δ*myr1* cLuc GFP⁺	[4]
T. gondii RH Δrop17 cLuc GFP⁺	[4]
T. gondii ME49 RFP	[4]
T. gondii RH Δ*tgwip* GFP⁺	[5]
T. gondii Pru Δ*hpt* GFP⁺ (PRU A7)	[6]
T. gondii Pru Δ*hpt* GFP⁺ Δ*gra15*	[7]
T. gondii RH Δ*hpt*	[8]
T. gondii RH Δ*hpt* Δ*rop16*	[8]
T. gondii Pru Δ*ku80*	[9]
T. gondii Pru Δku80 Δ*myr1*	[9]
T. gondii Pru Δ*ku80* Δ*tgist*	[10]
T. gondii PruΔku80 Δ*gra28*	[11]
T. gondii RHΔku80 Δgra24	[12]
T. gondii CPS mCherry	[13,14]
qPCR murine primers	Sequences 5'-3'
*GAPDH*_Forward	TGACCTCAACTACATGGTCTACA
*GAPDH*_Reverse	CTTCCCATTCTCGGCCTTG
*Ipo8*_Forward	CTATGCTCTCGTTCAGTATGC
*Ipo8*_Reverse	GTCCGAAAGATCTCCATCCA
*Tbp*_Forward	GGGGAGCTGTGATGTGAAGT
*Tbp*_Reverse	CCAGGAAATAATTCTGGCTCA
*Gad67*_Forward	GTGACCAGGGTGCCCGCTTC
*Gad67*_Reverse	TGCGCAGTTTGCTCCTCCCC
*Gad65*_Forward	GCTGGAACCACCGTGTATGG
*Gad65*_Reverse	TCCACGTGCATCCAGATCTTAT
*HTR3A*_Forward	TCAATGAGTTCGTGGATGTG
*HTR3A*_Reverse	TGTAGTTCTGAACTTCGCC
*NKCC1*_Forward	GATGCTGTGGTCGCATACACT
*NKCC1*_Reverse	CAGCGGACTAATACACCCT
*Cav1.3*_Forward	TGCACAGATGAAGCCAAAAG
*Cav1.3*_Reverse	GACCAACGTTCTCACCGTTT
qPCR human primers	Sequences 5'-3'
*Ipo8*_Forward	CGAAGCTCACTAGTTTTGACCC
*Ipo8*_Reverse	GCAAAGGAAGGGGAATTGA
*Tbp*_Forward	TCTGGGTTTGATCATTCTGTAG
*Tbp*_Reverse	GAGCTGTGATGTGAAGTTTCC
Angpt2_Forward	TGGCCGCAGCCTATAACA
Angpt2_Reverse	TCTCTGGCAGGAGGAAAGTGT

1. Barragan, A. & Sibley, L. D. Transepithelial migration of *Toxoplasma gondii* is linked to parasite motility and virulence. *J. Exp. Med.* **195**, 1625–1633 (2002).
2. Kim, K., Eaton, M. S., Schubert, W., Wu, S. & Tang, J. Optimized expression of green fluorescent protein in *Toxoplasma gondii* using thermostable green fluorescent protein mutants. *Mol. Biochem. Parasitol.* **113**, 309–313 (2001).

3. Boyle, J. P., Saeij, J. P. J. & Boothroyd, J. C. *Toxoplasma gondii*: inconsistent dissemination patterns following oral infection in mice. *Exp. Parasitol.* **116**, 302–305 (2007).
4. Wang, Y. *et al.* Three *Toxoplasma gondii* Dense Granule Proteins Are Required for Induction of Lewis Rat Macrophage Pyroptosis. *mBio* **10**, e02388-18 (2019).
5. Sangaré, L. O. *et al.* In Vivo CRISPR Screen Identifies TgWIP as a *Toxoplasma* Modulator of Dendritic Cell Migration. *Cell Host Microbe* **26**, 478-492.e8 (2019).
6. Kim, S.-K., Karasov, A. & Boothroyd, J. C. Bradyzoite-specific surface antigen SRS9 plays a role in maintaining *Toxoplasma gondii* persistence in the brain and in host control of parasite replication in the intestine. *Infect. Immun.* **75**, 1626–1634 (2007).
7. Mukhopadhyay, D., Arranz-Solís, D. & Saeij, J. P. J. *Toxoplasma* GRA15 and GRA24 are important activators of the host innate immune response in the absence of TLR11. *PLoS Pathog.* **16**, e1008586 (2020).
8. Jensen, K. D. C. *et al.* *Toxoplasma* polymorphic effectors determine macrophage polarization and intestinal inflammation. *Cell Host Microbe* **9**, 472–483 (2011).
9. Braun, L. *et al.* The *Toxoplasma* effector TEEGR promotes parasite persistence by modulating NF-κB signalling via EZH2. *Nat. Microbiol.* **4**, 1208–1220 (2019).
10. Gay, G. *et al.* *Toxoplasma gondii* TgIST co-opts host chromatin repressors dampening STAT1-dependent gene regulation and IFN-γ-mediated host defenses. *J. Exp. Med.* **213**, 1779–1798 (2016).
11. Ten Hoeve, A. L. *et al.* The *Toxoplasma* effector GRA28 promotes parasite dissemination by inducing dendritic cell-like migratory properties in infected macrophages. *Cell Host Microbe* **30**, 1570-1588.e7 (2022).
12. Braun, L. *et al.* A *Toxoplasma* dense granule protein, GRA24, modulates the early immune response to infection by promoting a direct and sustained host p38 MAPK activation. *J. Exp. Med.* **210**, 2071–2086 (2013).
13. Fox, B. A. & Bzik, D. J. De novo pyrimidine biosynthesis is required for virulence of *Toxoplasma gondii*. *Nature* **415**, 926–929 (2002).
14. Konradt, C. *et al.* Endothelial cells are a replicative niche for entry of Toxoplasma gondii to the central nervous system. *Nat. Microbiol.* **1**, 1–8 (2016).

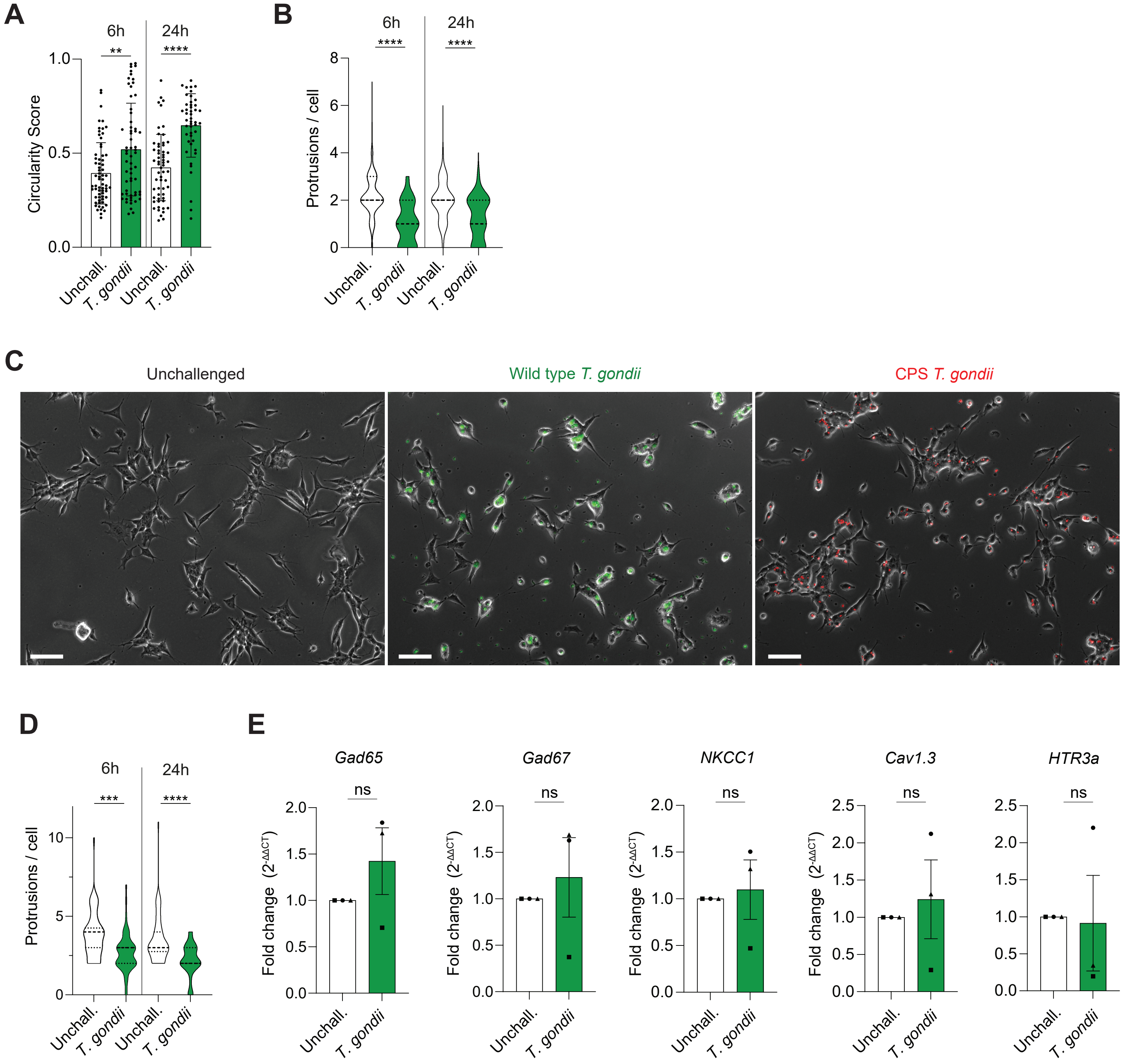

Supplementary Figure S1: Impact of *T. gondii* infection on SH-SY5Y morphology

(**A**) Mean cell body circularity (±SEM) scored for undifferentiated SH-SY5Ycells, unchallenged or challenged with GFP-expressing *T. gondii* (RH-LDM) for 6-24 h. Each data point represents one cell body (n = 3).

(**B**) Number of protrusions for undifferentiated SH-SH5Y cells treated as in (A). Dotted lines represent quartiles and bold lines medians (n = 3).

(**C**) Representative micrographs of undifferentiated SH-SY5Y cells, unchallenged, challenged with GFP-expressing wild type *T. gondii* (RH-LDM), or challenged with mCherry expressing *T. gondii* CPS (RH-CPS) for 24 h. Scale bar: 100 µm.

(**D**) Number of protrusions for differentiated SH-SH5Y cells, unchallenged or challenged with GFP-expressing *T. gondii* (RH-LDM) for 6-24 h (n = 3).

(**E**) qPCR analyses of cDNA from primary cortical neurons challenged with *T. gondii* (RH-LDM) for 24 h. Transcription of GABA synthesis enzymes Gad65, Gad67, Na-K-Cl cotransporter NKCC1, voltage-gated calcium channel Cav1.3 and and serotonine receptor HTR3a were assessed. qPCR data are displayed as fold change ($2^{-\Delta\Delta Ct}$) in relation to unchallenged condition (n = 3).

All data are from 3 independent experiments with 50-150 cells analysed per condition for each experiment. Statistical analyses were performed with (A-D) ANOVA, (E) Student's t-test, ** P < 0.005, *** P < 0.0005, **** P < 0.0001, ns: non-significant.

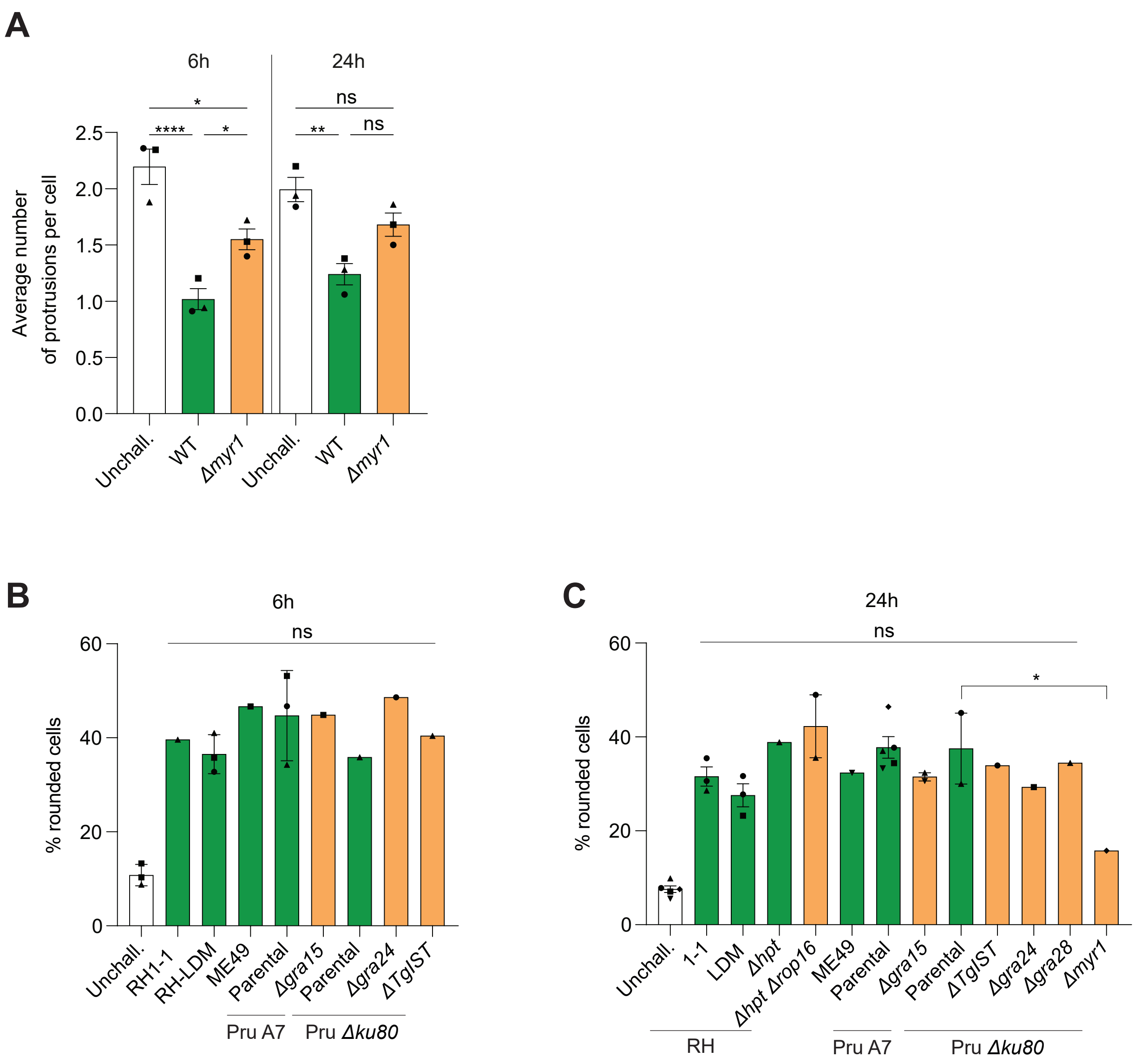

Supplementary Figure S2: Screenings of *T. gondii* mutants for effects on SH-SY5Y cell morphology

(**A**) Average number of protrusions for undifferentiated SH-SH5Y cells, unchallenged or challenged with either *T. gondii* wild type (RH-LDM) or *Δmyr1* for 6-24 h (n = 3).

(**B**, **C**) Percentage of cells (mean ± SEM) with rounded cell morphology related to the total cell count. SH-SY5Y were challenged (A) with RH1-1, RH-LDM, ME49, Pru A7, Pru A7*Δgra15*, Pru A7*Δku80*, Pru A7*Δku80 Δgra24*, Pru A7*Δku80 ΔTgIST* for 6 h and (B) with RH1-1, RH-LDM, RH *Δhpt*, RH *Δhpt Δrop17*, ME49, Pru A7, Pru A7*Δ gra24*, Pru A7*Δku80*, Pru A7*Δku80 ΔTgIST*, Pru A7*Δku80 Δgra24*, Pru A7*Δku80 Δgra28*, for 24 h. Parental strain indicates *T. gondii* strain used to generate the associated mutant.

Each data point represents one biological replicate with 50-100 cells counted/data point; (n = 1-3). ANOVA * $P < 0.05$, ** $P < 0.005$, **** $P < 0.0001$, ns: non-significant.

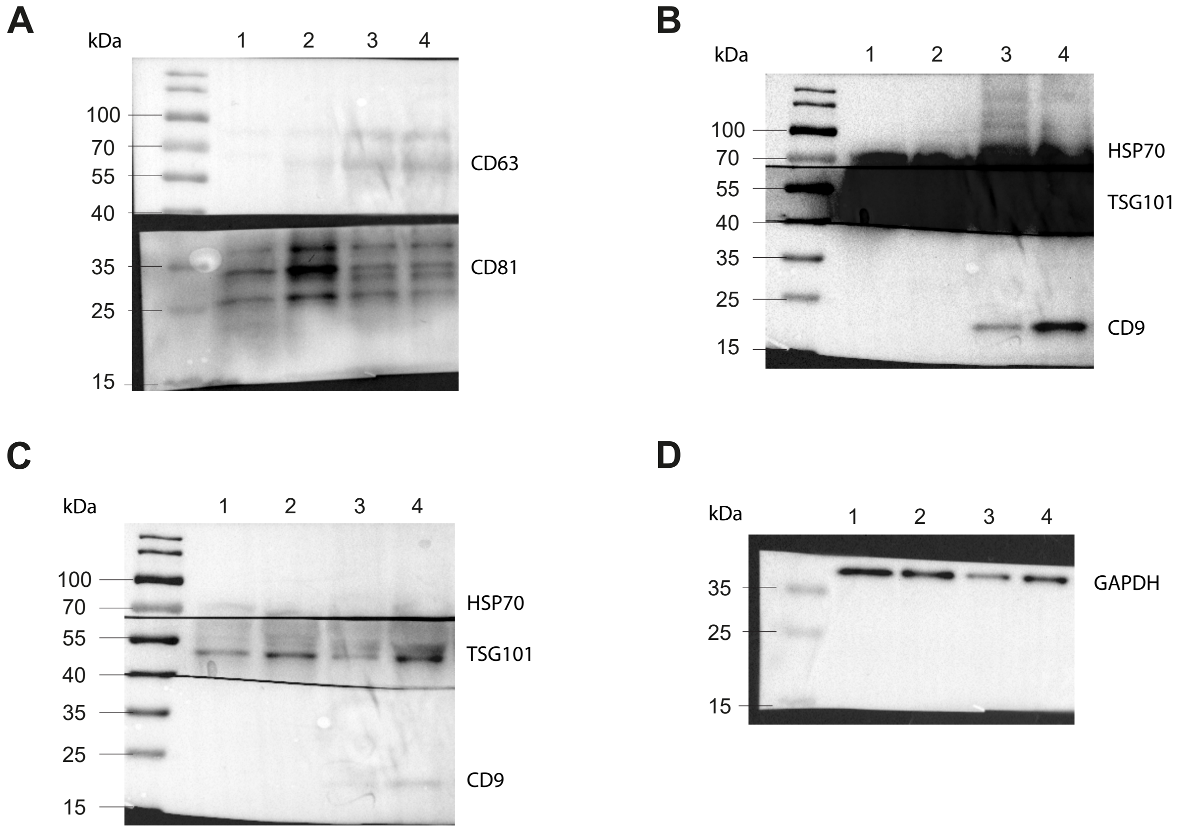

Supplementary Figure S3: Original Western blots

(**A**, **B**, **C**, **D**) Samples were immunoblotted for EV markers as indicated under Methods (A) CD63 and CD81, (B, C) HSP70, TSG101 and CD9, (D) GAPDH as loading control.

Lanes: (1) lysate from 10 000 SH-SY5Y cells, (2) lysate from 30 000 SH-SY5Y cells, (3) EV-enriched fraction from supernatant of unchallenged SH-SY5Y cells and (4) EV-enriched fraction from supernatant of *T. gondii*-challenged SH-SY5Y cells. Equal amounts were loaded in lanes 3 and 4, equivalent to supernatants collected from $5x10^6$ SH-SY5Y cells. CD81 and HSP70 markers were not used in this study. Blots are representative of multiple experiments.
