# Supplementary material for: Cytoskeletal alterations in neuronal cells implicate Toxoplasma gondii secretory machinery and host microRNA-containing extracellular vesicles
Source: Sci Rep. 2025 Apr 12;15:12606. doi: 10.1038/s41598-025-96298-8 (PMC11993698; doi:10.1038/s41598-025-96298-8)
Supplement: Supplementary file 4 — Supplementary Material 4 [file 41598_2025_96298_MOESM4_ESM.pdf]

# Description of additional supplementary files

## Supplementary Movie S1: Morphology of unchallenged and *T. gondii challenged* SH-SY5Y cells

Representative time lapse videos of unchallenged SH-SY5Y cells (upper panel) and SH-SY5Y cells challenged with GFP-expressing *T. gondii* tachyzoites (RH-LDM, MOI 7; lower panel). The relatively high MOI was used in order to visualize invasion and infected cell events in a given field of view. Time lapses were taken from 1 h to 6 h post-challenge and images were taken every 3 min. Scale bar: 20 µm.

## Supplementary Movie S2: EV particles in supernatants from *T. gondii*-challenged SH-SY5Y cells visualized by NTA

Representative video shows real-time high-resolution nanoparticle detection in cell supernatant fractions enriched for EVs as indicated under Materials and Methods. Video captures light scattering by particles moving under Brownian motion.
